# Para-Aortic Nodal Radiation in the Definitive Management of Node-Positive Cervical Cancer

**DOI:** 10.3389/fonc.2021.664714

**Published:** 2021-04-29

**Authors:** Jason C. Sanders, Donald A. Muller, Sunil W. Dutta, Taylor J. Corriher, Kari L. Ring, Timothy N. Showalter, Kara D. Romano

**Affiliations:** ^1^ Department of Radiation Oncology, University of Virginia School of Medicine, Charlottesville, VA, United States; ^2^ Division of Gynecologic Oncology, Department of Obstetrics and Gynecology, University of Virginia School of Medicine, Charlottesville, VA, United States

**Keywords:** cervical cancer, radiation therapy, brachytherapy, para-aortic nodal irradiation, chemoradiation (CRT)

## Abstract

**Objectives:**

To investigate the safety and outcomes of elective para-aortic (PA) nodal irradiation utilizing modern treatment techniques for patients with node positive cervical cancer.

**Methods:**

Patients with pelvic lymph node positive cervical cancer who received radiation were included. All patients received radiation therapy (RT) to either a traditional pelvic field or an extended field to electively cover the PA nodes. Factors associated with survival were identified using a Cox proportional hazards model, and toxicities between groups were compared with a chi-square test.

**Results:**

96 patients were identified with a mean follow up of 40 months. The incidence of acute grade ≥ 2 toxicity was 31% in the elective PA nodal RT group and 15% in the pelvic field group (Chi-square p = 0.067. There was no significant difference in rates of grade ≥ 3 acute or late toxicities between the two groups (p>0.05). The KM estimated 5-year OS was not statistically different for those receiving elective PA nodal irradiation compared to a pelvic only field, 54% *vs.* 73% respectively (log-rank p = 0.11).

**Conclusions:**

Elective PA nodal RT can safely be delivered utilizing modern planning techniques without a significant increase in severe (grade ≥ 3) acute or late toxicities, at the cost of a possible small increase in non-severe (grade 2) acute toxicities. In this series there was no survival benefit observed with the receipt of elective PA nodal RT, however, this benefit may have been obscured by the higher risk features of this population. While prospective randomized trials utilizing a risk adapted approach to elective PA nodal coverage are the only way to fully evaluate the benefit of elective PA nodal coverage, these trials are unlikely to be performed and instead we must rely on interpretation of results of risk adapted approaches like those used in ongoing clinical trials and retrospective data.

## Introduction

The use of definitive chemoradiation in the management of locally advanced cervical cancer has been well validated (1–5) and remains the standard of care for these patients (6). However, the management of para-aortic (PA) lymph nodes in these patients has remained controversial. The decision to use an extended field technique is commonplace for those with involved PA lymph nodes; however, the use of elective PA fields is less clear in patients with clinically negative PA lymph nodes. In practice, elective PA coverage has been considered for those with multiple positive pelvic lymph nodes, positive common iliac lymph nodes, uterine fundal involvement, or bulky primary tumors which may disrupt the normal lymphatic drainage (6–9).

Initial support for the use of an extended field technique came from the randomized Radiation Therapy Oncology Group (RTOG) 79-20, which showed an overall survival (OS) and distant metastasis advantage with elective PA coverage compared to traditional whole pelvis fields in the absence of chemotherapy (10, 11). Conversely, results from the European Organization for Research and Treatment of Cancer (EORTC) showed only a potential decrease in isolated PA nodal failures with an extended field technique, but no differences in local control, distant metastases, or overall survival (12). In the era of chemotherapy for cervical cancer, results from RTOG 92-10 raised concerns over significant toxicity in patients with involved PA nodes treated using extended fields with twice daily radiation with concurrent chemotherapy in the 2D era (13). As a result, RTOG 90-01 compared concurrent chemoradiation with a whole pelvic field to an extended field without chemotherapy and found that the survival benefit seen in 79-20 disappeared and actually favored the whole pelvic field chemoradiation arm (14, 15).

Modern techniques utilizing 3D conformal radiation therapy, intensity-modulated radiation therapy (IMRT), and image-guided radiation therapy (IGRT) are expected to have reduced acute and late toxicities, however, no randomized studies have assessed the outcomes between whole pelvic versus extended field techniques utilizing these techniques with concurrent chemotherapy. In this analysis we sought to compare the outcomes of extended field PA nodal irradiation and traditional pelvic fields at our institution utilizing modern treatment techniques.

## Materials and Methods

### Patient Selection

Under an institutional review board approved protocol, patients with locally advanced cervical cancer treated with definitive radiation between 2004 and 2017 at the University of Virginia were retrospectively reviewed. Patients were staged using the 2009 International Federation of Gynecology and Obstetrics (FIGO) staging criteria (16), however, details regarding clinically positive pelvic lymph node metastases were also recorded. Patients with clinically staged FIGO IB-IVA stages with involved pelvic lymph nodes were included (currently, considered FIGO IIIC1); those with clinically positive PA lymph nodes and those with metastatic disease beyond the PA lymph nodes were excluded. Clinical staging and lymph node evaluation were based on exam and imaging at the time of diagnosis and treatment, and were not retrospectively assigned. Lymph nodes were considered clinically positive if they were 1 cm or larger in short axis or reported as pathologically enlarged on computed tomography or if they were reported as pathologic on PET/CT (typically SUV >4 with consideration of CT-based size and morphology). Patients were required to have a pathologically confirmed diagnosis of cervical cancer, including any histology other than small cell carcinoma. Patients who received external beam radiation (EBRT) at an outside facility were included, as long as they received brachytherapy at the primary institution, and details of their EBRT were available for review. Clinical prognostic factors were collected, including: age, smoking status, medical comorbidities, Eastern Cooperative Oncology Group (ECOG) performance status, tumor histology, clinical tumor size, lymph node involvement and location, and FIGO stage.

### Treatment

All patients received both EBRT and brachytherapy for their cervical cancer. EBRT encompassed a standard pelvic field using traditionally fractionated radiation (1.8 to 2 Gy/fx) to a dose of 45 to 50.4 Gy with consideration of a parametrial or nodal boost at the discretion of the treating radiation oncologist. Elective PA nodal RT was performed at the discretion of the treating radiation oncologist, but generally was considered for uterine fundus involvement, multiple positive pelvic nodes, positive common iliac nodes, and/or bulky primary tumors. Elective PA nodal RT was prescribed to 45 to 50.4 Gy. The superior border of the elective PA nodal field was typically defined as the level of the renal vessels on CT-based planning, but was left at the discretion of the treating physician based on individual anatomy and patient risk factors. The superior border of the pelvic only field was defined as the top of the common iliac vessels on CT-based planning. 3D conformal radiation and IMRT techniques were both allowed for the EBRT component. Patients treated from 2004 to the first half of 2009 received low dose rate (LDR) brachytherapy with one to two implants of a Cesium-137 source. High dose rate (HDR) brachytherapy was phased in starting in 2009, with patients receiving four to six implants with an Iridium-192 source delivered using a remote afterloader. All patients treated with HDR brachytherapy were treated with the generation of 3D imaged guided brachytherapy, while those receiving LDR brachytherapy were treated with traditional 2D planning. Concurrent weekly Cisplatin at 40 to 50 mg/m^2^ was administered concurrently with external beam radiation with a target of five to six cycles. Dose reduction, discontinuation, or omission of chemotherapy was at the discretion of the treating gynecologic oncologist based on consideration of performance status, medical comorbidities, absolute neutrophil count, absolute platelet count, and patient tolerance.

### Follow-Up

Following completion of radiation, patients underwent routine evaluation by the treating Gynecologic Oncologist and/or Radiation Oncologist every 3 months for the first 2 years, at least every 6 months up to 5 years post treatment, then yearly beyond 5 years after treatment. Length of follow up was calculated from the date of completion of radiation to the date of most recent oncologic follow up or date of death. Recurrences were based on clinical exam, biopsy, or surveillance imaging, and were classified as local (within the cervix, vagina, parametria, or surrounding tissues), regional (within pelvic lymph nodes), PA (within PA lymph nodes), or distant (within lymph nodes beyond the PA nodes or other organs). Treatment related toxicities were graded according to the Common Terminology Criteria for Adverse Events (CTCAE) version 5.0 (17).

### Statistical Analysis

Kaplan-Meier (KM) method was used to estimate disease free survival (DFS), distant metastasis free survival (DMFS), and overall survival (OS). Univariable analyses (UVA) and multivariable analysis (MVA) using Cox proportional hazards model were performed to identify prognostic factors and hazard ratios (HR) with 95% confidence intervals (CI) for OS, DMFS, and DFS. Factors identified with a trend towards association (p <0.10) on UVA were included in the MVA, with p <0.05 considered statistically significant. Patients were classified as either squamous cell or non-squamous cell on univariable and multivariable analyses for DFS, DMFS, and OS. Toxicities between the two groups were compared using a chi-squared test, with p <0.05 considered statistically significant.

## Results

### Patient Characteristics

96 consecutive patients were identified who met the selection criteria, with a mean follow up of 40 months and mean patient age of 47 years (range, 26–73 years). Squamous cell carcinoma was the most frequent histology (88%, n = 84), while adenocarcinoma (8%, n = 8) and adenosquamous (3%, n = 3) histologies were less common. Clinically involved common iliac nodal involvement was less common (18%, n = 12), and was more frequent in those receiving elective PA coverage (**Table 1**). Most patients had a pre-treatment PET/CT (n = 173, 65%) and/or MRI (n = 194, 73%) prior to starting radiation.

**Table 1:** Clinical, disease, and treatment characteristics of 96 patients with node positive cervical cancer treated with definitive intent radiation.

	Pelvic Field Only (n = 47)		Elective PA Field (n = 49)		p-value
	No. of Patients	%	No. of Patients	%	
**Clinical characteristics**					
Age (years old, median, range)	47	31-72	45	26-73	0.45
Smoking history					0.77
No smoking history	23	49	21	43	
Former smoker	9	19	9	18	
Current smoker	15	32	19	39	
Diabetes	6	13	3	6	0.26
ECOG					0.08
0	22	47	32	70	
1	18	38	12	26	
2	7	15	2	4	
**Disease characteristics**					
Primary tumor size (cm, median, range)	5	1–14	6	2–11	0.36
FIGO stage					0.25
IB1	7	15	4	8	
IB2	12	26	14	29	
IIA2	0	0	3	6	
IIB	16	34	12	24	
IIIB	12	25	14	29	
IVA	0	0	2	4	
No. involved lymph nodes					0.07
1	24	51	13	26	
2	12	26	15	31	
3+	11	23	21	43	
Lymph node location					0.001
Low pelvic	45	96	34	69	
Common Iliac	2	4	15	31	
Histology					
Squamous cell	41	87	43	88	0.61
Adenocarcinoma	3	6	5	10	
Adenosquamous	2	4	1	2	
Neuroendocrine	1	2			
**Treatment characteristics**					
Concurrent chemotherapy	45	96	48	98	0.30
Brachytherapy	47	100	49	100	0.90
LDR	11	23	28	57	0.53
HDR	36	77	21	43	0.001

PA, para-aortic; No, number; ECOG, Eastern Cooperative Oncology Group; FIGO, International Federation of Gynecology and Obstetrics; LDR, low dose rate; HDR, high dose rate.

### Treatment Parameters

Patients were relatively evenly split between treatment field size, with 51% receiving elective PA coverage and 49% receiving a traditional pelvic field. The majority of patients (60%, n = 57) were treated with HDR brachytherapy, with the remainder of the patients receiving LDR brachytherapy (40%, n = 39). Those treated with elective PA coverage were more likely to receive LDR brachytherapy compared to those who were treated with a traditional pelvic field (57% vs. 23%, p = 0.001). Concurrent chemotherapy was administered to 97% of patients, but was excluded due to performance status and/or medical comorbidities for three patients (**Table 1**).

### Outcomes

**Figure 1:**
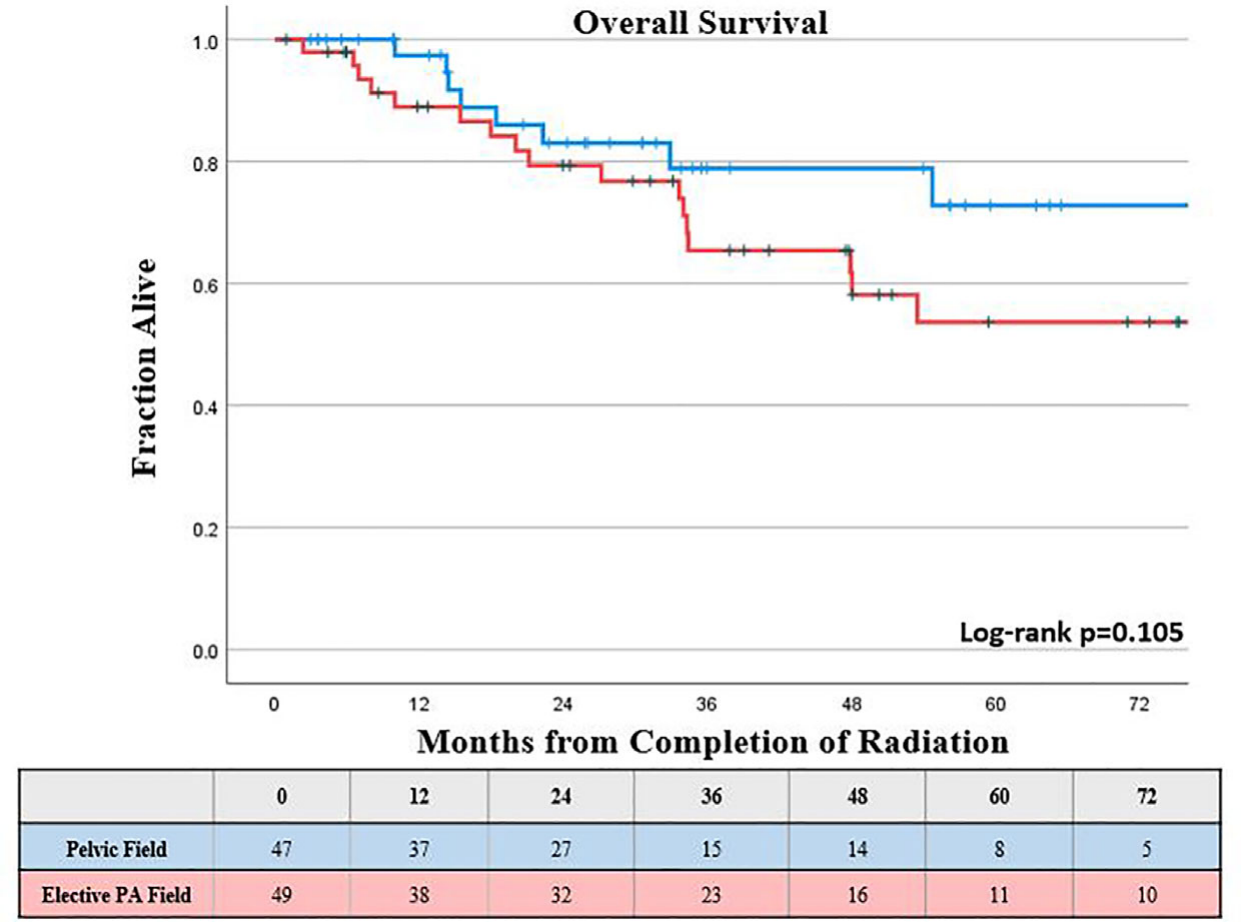
Kaplan-Meier estimated overall survival by type of treatment field.

The KM estimated 5-year OS was slightly lower, but not significantly different for those who received elective PA nodal RT compared to a pelvic field, 54% *vs.* 73%, respectively (log-rank p = 0.11; **Figure 1**). On univariable analysis, increasing FIGO Stage, clinically involved common iliac lymph nodes, and increasing primary tumor size were associated with worse overall survival (p <0.05). On multivariable analysis, the presence of clinically positive common iliac lymph nodes (HR, 2.66 compared to lower pelvic node involvement; CI, 1.01–6.99; p = 0.048) was the only factor associated with worse OS (**Table 2**).

**Table 2:** Analysis of factors associated with overall survival, distant metastasis free survival, and disease-free survival for 96 patients with node-positive cervical cancer treated with definitive intent radiation.

	Overall Survival				Distant Metastasis-Free Survival				Disease-Free Survival			
	UVA	MVA			UVA	MVA			UVA	MVA		
	P	P	HR	CI	P	P	HR	CI	P	P	HR	CI
**Clinical characteristics**												
Age (years old)*	0.96				0.77				0.67			
History of diabetes	0.47				0.37				0.29			
Smoking history	0.62				0.68				0.42			
ECOG	0.43				0.12				0.35			
**Disease characteristics**												
FIGO stage*	**0.01**	0.172	1.17	0.93–1.46	**0.004**	0.081	1.21	0.98–1.49	**0.002**	**0.002**	1.32	1.11–1.56
Common iliac node +	**0.04**	**0.048**	2.66	1.01–6.99	0.15				0.11			
≥3 Involved nodes	0.65				0.96				0.57			
Tumor size (cm)*	**0.04**	0.281	1.12	0.92–1.36	0.095	0.61	1.05	0.87–1.28	0.13			
Tumor histology	0.53				0.37				0.56			
Elective PA nodal Coverage	0.11				0.26				0.26			

UVA, univariable; MVA, multivariable; P, p-value, bolded for p < 0.05; HR, hazard ratio; 95% CI, 95% confidence interval; +, positive; ECOG, Eastern Cooperative Oncology Group; FIGO, International Federation of Gynecologic and Obstetrics; PA, para-aortic.

*Analyzed as a continuous variable.

**Figure 2:**
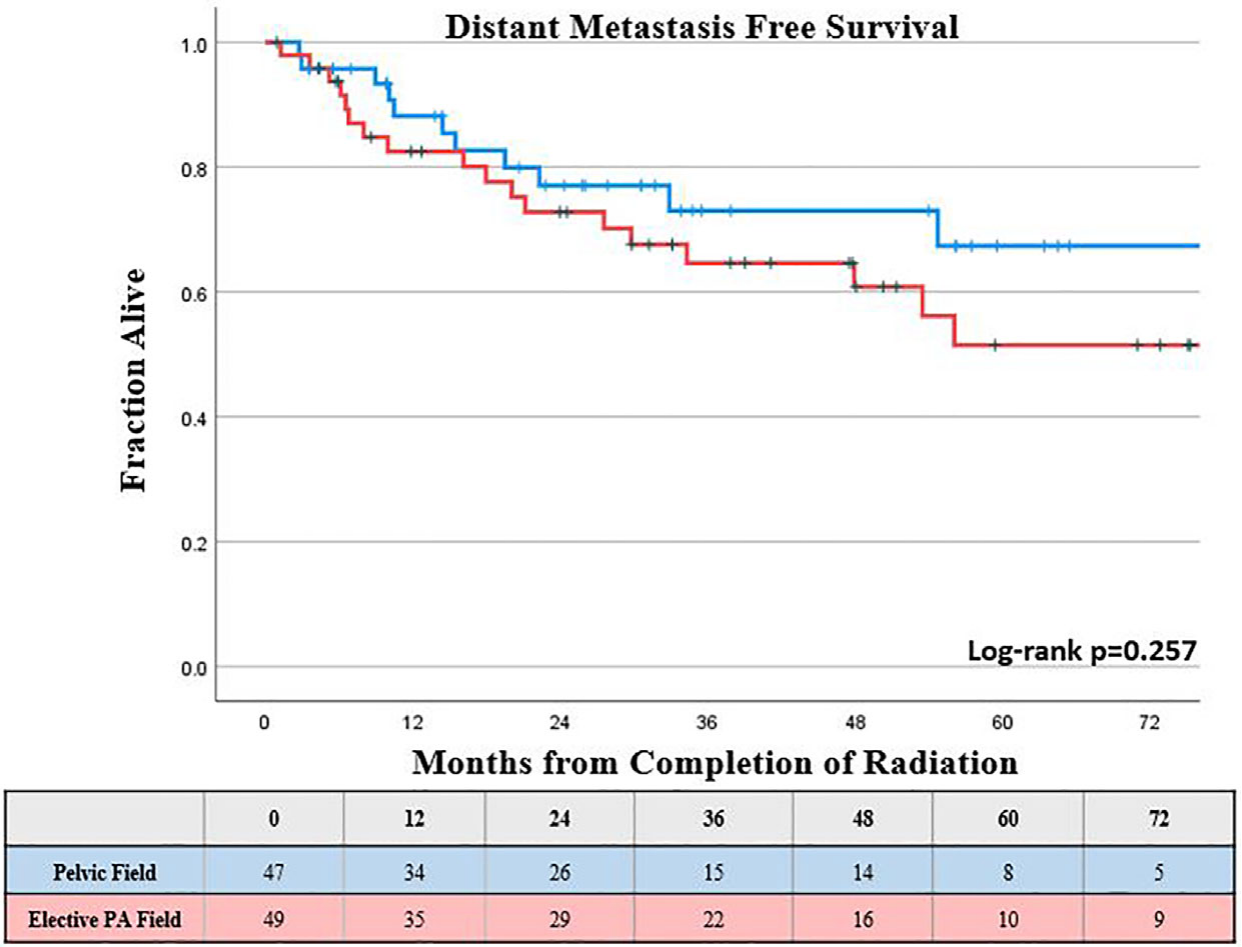
Kaplan-Meier estimated distant metastasis free survival by type of treatment field.

**Figure 3:**
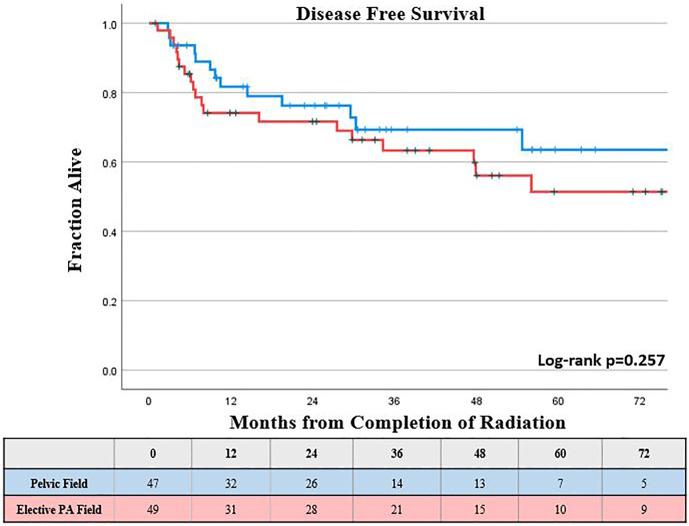
Kaplan-Meier estimated disease free survival by type of treatment field.

The KM estimated 5-year DMFS patients was slightly lower, but not statistically different for those who received elective PA nodal RT compared to a pelvic field, 52% *vs* 67%, respectively (log-rank p = 0.26; **Figure 2**). Increasing FIGO stage was associated with worse DMFS on univariable analysis (p < 0.05), while there was a trend towards worse DMFS with increasing primary tumor size (p <0.1); however, these associations were not significant for DMFS on multivariable analysis (**Table 2**). The KM estimated 5-year DFS was slightly lower, but not significantly different for those who received elective PA nodal RT compared to a pelvic field, 51% *vs.* 64% respectively (log-rank p = 0.26; **Figure 3**). On both UVA and MVA, increasing FIGO Stage (HR, 1.32 per stage increase; CI, 1.11–1.56; p = 0.002) was the only identified factor associated with worse DFS (**Table 2**).

PA nodal failures occurred in only 7 patients (7.3%), with 6 of these occurring in those who did not receive PA nodal RT. Only two of these patients had isolated PA nodal failures, while four had synchronous distant metastases and one had a synchronous local failure. The median time for PA nodal failure was 19 months (range, 3–56 months), with isolated PA nodal recurrences occurring at 10 and 30 months following treatment. Both isolated PA nodal failures occurred in patients who had a single involved lower pelvic (not common iliac) lymph node and did not receive elective PA nodal RT. These two patients were subsequently salvaged with chemoradiation. Following salvage therapy, one patient was alive and free of disease at the time of last follow up, and 1 patient experienced a subsequent fatal local recurrence of her primary tumor 6 months following salvage PA nodal irradiation.

There were no instances of acute grade 5 toxicity. The incidence of acute grade 2+ toxicity was 31% in the elective PA nodal RT group and 15% in the pelvic field group (Chi-square p = 0.067). However, the incidence of acute grade 3+ toxicity was 14% in the elective PA nodal RT group and 6% in the pelvic field group (Chi-square p = 0.205). There were no late grade 4 or 5 toxicities. The rate of late grade 3 toxicity was 8% in the elective PA nodal RT group and 9% in the pelvic field group (Chi-square p = 0.926).

## Discussion

Concurrent chemoradiation with brachytherapy boost is currently the standard of care for locally advanced cervical cancer. However, practices vary regarding the inclusion of elective extended field PA nodal RT in the setting of clinically uninvolved PA nodes. The original studies demonstrating a benefit to elective PA fields did not include chemotherapy, and there is currently no randomized data utilizing modern treatment techniques to support either inclusion or exclusion of an elective PA field for high risk patients (10, 11). The EMBRACE study demonstrated that while most of the nodal disease at diagnosis was located in the pelvis, the majority of nodal failures were in the PA nodal region (18). Additionally, we know that there are limitations to the sensitivity and specificity of clinical staging. Ramirez et al. prospectively enrolled patients with clinically negative PA lymph nodes on PET/CT to laparoscopic extraperitoneal PA lympadenectomy and found that rates of involved PA lymph nodes at surgery were 12% in node negative patients and up to 22% in those with involved pelvic lymph nodes (19).

Extended field elective PA nodal RT with older treatment techniques was historically discounted over concerns for increased toxicity (13); however, modern series utilizing advanced treatment techniques have shown that inclusion of a PA nodal field does not increase significant toxicities and can be performed safely in patients with cervical cancer (20, 21). Despite studies showing the safety and feasibility of extended PA nodal fields, only a limited number of small population, single institution studies supporting (9) or arguing against (7) elective PA coverage exist. The currently open EMBRACE II protocol incorporates a risk-adapted model for the selective use of PA nodal irradiation for high risk patients, and we await those results (8). In the interim, we report our experience with elective PA nodal RT utilizing modern treatment techniques and concurrent chemotherapy in clinically node-positive patients.

This study demonstrates that elective PA nodal RT is safe and feasible but is not associated with improved disease specific outcomes for patients with clinically positive pelvic lymph nodes. At our institution, elective PA nodal RT has been utilized at the discretion of the treating physician for higher risk patients. Factors leading toward the inclusion of elective PA nodal RT have historically been large primary tumor, uterine fundal involvement, multiple positive pelvic nodes, or common iliac nodal involvement. This preference is reflected in our data as elective PA nodal RT patients were more likely to have: ≥3 clinically positive lymph nodes (43% vs 23%), involved common iliac nodes (31% *vs.* 4%), and slightly larger primary tumors (median 6.0 cm *vs.* 5.0 cm) compared to their pelvic field counterparts. The higher risk features of these patients may have diminished any potential survival advantages that an extended PA field may have provided. In this study, we found that the presence of common iliac nodes was associated with worse OS on univariable and multivariable analysis. As such, a risk adapted approach, similar to that used in the EMBRACE II protocol (8), is likely to optimize the potential benefit from elective PA nodal RT. Additionally, patients who received elective PA nodal coverage were less likely to be treated with 3D image guided brachytherapy compared to those who received pelvic only fields (43% *vs.* 77%, p = 0.001) which may also impact interpretation of the outcomes between these groups.

Salvage options for isolated PA nodal failures exist, including RT, surgery, and/or systemic therapy (6). In our study, PA nodal failures were rare (n = 7, 7.3%) and were usually (n = 5, 71%) associated with synchronous local and/or distant failures. Only two patients (2%) experienced isolated PA failures, neither of whom received elective PA nodal coverage in their initial treatment, and were successfully salvaged with concurrent chemoradiation to the PA lymph nodes. The question remains whether the five patients who developed concurrent PA and local/distant failures would have been spared with elective coverage of potentially micro-metastatic disease at the time of definitive treatment.

With respect to toxicity, there was no significant association between elective PA nodal RT and acute or late grade ≥ 3 toxicity. However, there was a trend towards an increased rate of acute grade 2+ toxicity with elective PA nodal RT compared to pelvic field RT (31% *vs*. 15%, p = 0.067). This possible increased incidence of non-severe acute toxicity, such as nausea and diarrhea, may be relevant to patient counseling when considering elective PA coverage.

There are limitations to this study including the retrospective nature with data from a single institution. Retrospective review may not fully capture the extent of toxicity, especially low grade toxicities. Support for increased toxicity with PA coverage has been well established in the 2D and 3D eras (13, 22); however, using modern planning techniques, such as IMRT, these differences seem to diminish (20). This study included patients treated with both 3D conformal and IMRT techniques, so the increased rate of non-severe acute toxicities might be overestimated by inclusion of those receiving 3D conformal radiation.

## Conclusion

To date there is no randomized evidence to guide the use of elective PA nodal RT in patients receiving definitive chemoradiation. Practices vary regarding its inclusion with original concerns over the increase in toxicity in the clinically PA node negative setting. In this large series of clinically node positive (current FIGO IIIC1) patients utilizing modern treatment techniques, we found that, elective PA nodal RT can safely be delivered without a significant increase in severe (grade 3+) acute or late toxicities, at the cost of a small increase in non-severe acute toxicities. In this series, there was no survival benefit observed with the receipt of elective PA nodal RT; however, this benefit may have been obscured by the higher-risk features of this population. While prospective randomized trials utilizing a risk adapted approach to elective PA nodal coverage are the only way to fully evaluate the benefit of elective PA nodal coverage, these trials are unlikely to be performed and instead we must rely on interpretation of results of risk adapted approaches like those used in ongoing clinical trials and retrospective data.

## Data Availability Statement

The data sets presented in this article are not readily available because patient level data not allowed to be shared per IRB. Requests to access the datasets should be directed to JS, jcs7em@hscmail.mcc.virginia.edu.

## Ethics Statement

The studies involving human participants were reviewed and approved by University of Virginia Institutional Review Board. Written informed consent for participation was not required for this study in accordance with the national legislation and the institutional requirements.

## Author Contributions

JS provided substantial contributions to this work *via* project conception, IRB submission, data acquisition, data analysis, data interpretation, manuscript drafting, and approval of the final manuscript version. DM provided substantial contributions to this work *via* data analysis, data interpretation, manuscript drafting, and approval of the final manuscript version. SD provided substantial contributions to this work *via* data acquisition, data analysis, data interpretation, manuscript drafting, and approval of the final manuscript version. TC provided substantial contributions to this work *via* data acquisition and approval of the final manuscript version. KRi provided substantial contributions to this work *via* data interpretation, manuscript drafting, and approval of the final manuscript version. TS provided substantial contributions to this work *via* project conception, IRB submission, data interpretation, manuscript drafting, and approval of the final manuscript version. KRo provided substantial contributions to this work *via* project conception, IRB submission, data interpretation, manuscript drafting, and approval of the final manuscript version. All authors contributed to the article and approved the submitted version.

## Conflict of Interest

The authors declare that the research was conducted in the absence of any commercial or financial relationships that could be construed as a potential conflict of interest.
